# GMP-like and MLP-like Subpopulations of Hematopoietic Stem and Progenitor Cells Harboring Mutated *EZH2* and *TP53* at Diagnosis Promote Acute Myeloid Leukemia Relapse: Data of Combined Molecular, Functional, and Genomic Single-Stem-Cell Analyses

**DOI:** 10.3390/ijms26094224

**Published:** 2025-04-29

**Authors:** Tal Shahar Gabay, Nofar Stolero, Niv Rabhun, Rawan Sabah, Ofir Raz, Yaara Neumeier, Zipora Marx, Liming Tao, Tamir Biezuner, Shiran Amir, Rivka Adar, Ron Levy, Noa Chapal-Ilani, Natalia Evtiugina, Liran I. Shlush, Ehud Shapiro, Shlomit Yehudai-Resheff, Tsila Zuckerman

**Affiliations:** 1 Hematology Research Center, Clinical Research Institute at Rambam, Rambam Health Care Campus, Haifa 3109601, Israel; talshahar77@gmail.com (T.S.G.); rawansabbah5@gmail.com (R.S.); s_yehudai@rambam.health.gov.il (S.Y.-R.); 2 The Ruth and Bruce Rappaport Faculty of Medicine, Technion, Haifa 3109601, Israel; 3 Department of Computer Science and Applied Mathematics, Weizmann Institute of Science, Rehovot 761001, Israel; ofir.raz@weizmann.ac.il (O.R.); yaara.neumeier@weizmann.ac.il (Y.N.); taoliming.too@gmail.com (L.T.); ronlevy6@gmail.com (R.L.); ehud.shapiro@weizmann.ac.il (E.S.); 4 Department of Immunology, Weizmann Institute of Science, Rehovot 761001, Israel; tcom111@gmail.com (T.B.); noa.chapal@weizmann.ac.il (N.C.-I.); liranshlush3@gmail.com (L.I.S.); 5 Department of Hematology and Bone Marrow Transplantation, Rambam Health Care Campus, Haifa 3109601, Israel

**Keywords:** acute myeloid leukemia (AML), relapse, hematopoietic stem and progenitor cells (HSPCs), single cell analysis (SCA), leukemogenic potential

## Abstract

Acute myeloid leukemia (AML) is associated with unfavorable patient outcomes primarily related to disease relapse. Since specific types of leukemic hematopoietic stem and progenitor cells (HSPCs) are suggested to contribute to AML propagation, this study aimed to identify and explore relapse-initiating HSPC subpopulations present at diagnosis, using single-cell analysis (SCA). We developed unique high-resolution techniques capable of tracking single-HSPC-derived subclones during AML evolution. Each subclone was evaluated for chemo-resistance, in vivo leukemogenic potential, mutational profile, and the cell of origin. In BM samples of 15 AML patients, GMP-like and MLP-like HSPC subpopulations were identified as prevalent at relapse, exhibiting chemo-resistance to commonly used chemotherapy agents cytosine arabinoside (Ara-C) and daunorubicin. Reconstruction of phylogenetic lineage trees combined with genetic analysis of single HSPCs and single-HSPC-derived subclones demonstrated two distinct clusters, originating from MLP-like or GMP-like subpopulations, observed both at diagnosis and relapse. These subpopulations induced leukemia development ex vivo and in vivo. Genetic SCA showed that these relapse-related subpopulations harbored mutated *EZH2* and *TP53*, detected already at diagnosis. This study, using combined molecular, functional, and genomic analyses at the level of single cells, identified patient-specific chemo-resistant HSPC subpopulations at the time of diagnosis, promoting AML relapse.

## 1. Introduction

Acute myeloid leukemia (AML) is an aggressive hematological malignancy with substantial inter- and intra-tumoral heterogeneity. Over the last decades, the understanding of the AML genetic makeup and its implications on the disease prognosis has significantly advanced and nowadays dictates the choice of appropriate therapeutic approaches [1,2]. The intra-patient phenotypic/genotypic diversity is a relatively new concept in AML, suggesting that in each patient, various subclones carry different genetic and non-genetic [3,4] compositions, potentially associated with their functional heterogeneity [5,6,7,8,9]. It is hypothesized that early leukemic hematopoietic stem and progenitor cells (HSPCs), enriched but not limited to a specific phenotype (e.g., CD34^+^CD38^−^), could be key players in AML maintenance and relapse [10,11]. The majority of adult AML patients will experience relapse within the first two years from diagnosis, which makes relapse prevention an important clinical challenge.

The HSPC involvement in relapse could be mediated by at least one of the three mechanisms leading to chemo-resistance, including the dormant nature of HSPCs, the evolutionary selection leading to the expansion of a unique clone, or the acquisition of additional mutations during the disease course [10,12]. Hence, it is conceivable that the characterization of relapse-initiating HSPCs at the time of diagnosis is crucial and may prevent disease progression after targeted treatment. Genomic analysis alone does not provide the full spectrum of HSPC features. This emphasizes the need for integrating genetic and functional analyses in both ex vivo and in vivo assessment of single leukemic cells derived from AML patients [7,8,13,14,15]. The diverse mutational profile of these cells may be one of the major factors promoting their survival in the highly competitive bone marrow (BM) niche [16]. The study by Potter et al., using genomic single-cell analysis (SCA) in the evaluation of samples obtained from AML patients carrying the *NPM1* mutation, has identified a number of branching subclones that had been undetectable in leukemic bulk cells and these specific subclones could be associated with disease relapse [17]. SCA is known to enable high-resolution evaluation of various cell subpopulations and thus provides an essential tool for the detection of specific leukemic cells responsible for future AML recurrence [9,16,18,19,20].

Yet, the inherent major limitation of SCA is associated with its inability to combine DNA analysis with functional assessments of such parameters as chemo-resistance and/or leukemogenic potential [8,16]. The current study aimed to identify and characterize oncogenic properties of the leukemic HSPC subpopulations observed at diagnosis that would ultimately drive AML relapse.

## 2. Results

### 2.1. Abnormal Distribution of HSPC Subpopulations in Samples Derived from MDS and AML Patients

**Figure 1:**
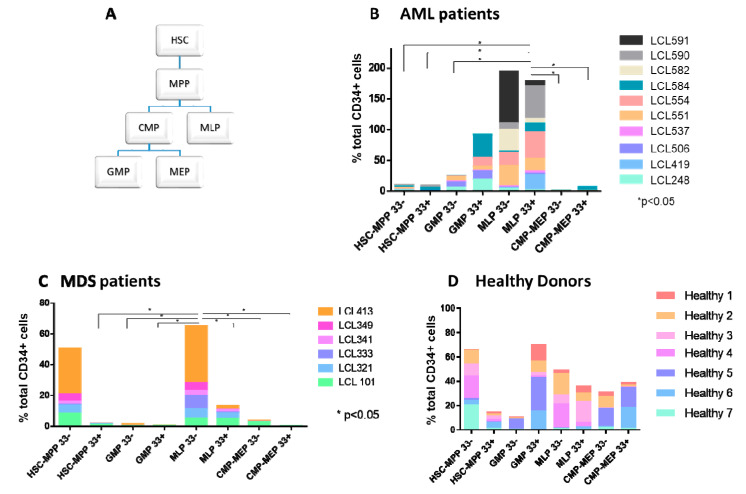
Hierarchy and distribution of leukemic (CD33^+^) and non-leukemic (CD33^−^) HSPC subpopulations. (**A**) Schematic presentation of the HSPC subpopulation hierarchy. (**B**) Distribution of leukemic (CD33^+^) and non-leukemic (CD33^−^) HSPCs in samples derived from AML patients. (**C**) Distribution of leukemic (CD33^+^) and non-leukemic (CD33^−^) HSPCs in samples derived from MDS patients. (**D**) Distribution of CD33^+^ and CD33^−^ HSPCs in samples derived from healthy donors. * *p* < 0.05. HSPC: hematopoietic stem and progenitor cell; HSC: hematopoietic stem cell; MPP: multipotent progenitor; CMP: common myeloid progenitor; MLP: multipotent lymphoid progenitor; GMP: granulocyte–monocyte progenitor; MEP: megakaryocyte–erythrocyte progenitor; MDS: myelodysplastic syndrome.

To explore whether the distribution and characteristics of leukemic HSPC subpopulations could change during AML progression and/or following chemotherapy, their composition at diagnosis, remission, and relapse was assessed. Immunophenotypic profiles of HSPC subpopulations were examined in BM samples of 10 newly diagnosed CD34^+^/CD38^−^ AML patients, 6 patients with myelodysplastic syndromes (MDS) (Table S1), and 7 healthy donors (HDs) (Figure 1A–D).

**Figure 2:**
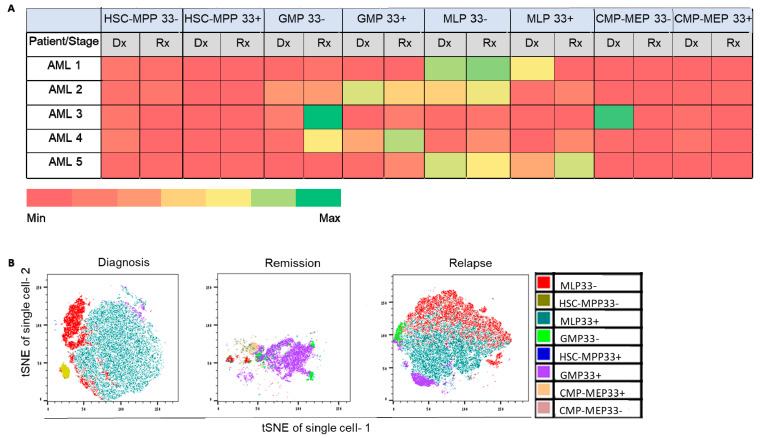
Major HSPC subpopulations involved in AML initiation, chemo-resistance, and relapse. (**A**) Percentage of AML patient HSPCs out of the total number of CD34^+^ cells at diagnosis and relapse. The colors represent the frequency of each HSPC subpopulation [ranging from minimum (red) to maximum (green)] in samples derived from five AML patients. (**B**) Distribution of HSPC subpopulations in the LCL440 patient, as assessed using the t-distributed stochastic neighbor embedding (t-SNE) tool, applied for the visualization of high-dimensional data. Each high-dimensional object is modeled by a two- or three-dimensional point in such a way that similar objects are modeled by nearby points and dissimilar objects are modeled by distant points with high probability. Dx: diagnosis; Rx: relapse; HSPC: hematopoietic stem and progenitor cell; HSC: hematopoietic stem cell; MPP: multipotent progenitor; CMP: common myeloid progenitor; MLP: multipotent lymphoid progenitor; GMP: granulocyte–monocyte progenitor; MEP: megakaryocyte–erythrocyte progenitor.

Unlike samples derived from AML and MDS patients (Figure 1B,C), those obtained from HDs exhibited the presence of all HSPC subpopulations, with no statistically significant difference between them (Figure 1D). Conversely, samples derived from MDS patients demonstrated significantly enlarged lymphoid lineage multipotent lymphoid progenitor 33^−^-like (MLP33^−^-like) and HSC-MPP33^−/+^-like subpopulations (*p* < 0.05). At AML diagnosis, the major HSPC subpopulations were MLP33^−/+^-like and myeloid lineage granulocyte/monocyte progenitor 33^+^-like (GMP33^+^-like). At AML relapse, the latter two subpopulations also prevailed (Figure 1B–D and Figure 2A,B), suggesting their potential role in disease progression. These data indicate an abnormal distribution of HSPC subpopulations in MDS and other leukemic states. Notably, CD33^+/−^ cells were found in samples derived from HDs, as well as from MDS and AML patients, which raises doubt regarding the specificity of CD33 as a leukemic marker (Figure 1A–D).

### 2.2. Involvement of Specific HSPC Subpopulations in Ex Vivo Chemo-Resistance and AML Recurrence

To study possible chemo-resistance of GMP33^+^-like, MLP33^+^-like, and HSC-MPP33^+^-like subpopulations, HSPCs derived from 10 AML patients were exposed to various concentrations of cytosine arabinoside (Ara-C) and daunorubicin. The GMP33^+^-like subpopulation was found to be resistant to both chemotherapy agents, even at their highest concentrations, whereas the MLP33^+^-like subpopulation was chemo-sensitive to both agents at any evaluated concentration (Figure S1A).

Our experiments demonstrated differential sensitivity to the above agents, with 20% of the patients being sensitive to Ara-C and resistant to daunorubicin, while another 20% showed a reverse pattern—resistant to Ara-C and sensitive to daunorubicin. In 30% of the patients, the aforementioned subpopulations were sensitive to both chemotherapy agents but did not completely disappear (Figure S1A), suggesting their potential role as a relapse source. In the remaining 30% of the patients, the two subpopulations were resistant to both drugs.

Further in-depth, representative, patient-specific chemo-resistance analyses of various HSPC subpopulations were performed in samples derived from two (LCL440, LCL465) of the 15 patients (Table S1; Figure S1B,C).

### 2.3. Phylogenetic Analysis of Single HSPCs and HSPC-Derived Subclones

To explore whether specific single AML HSPCs and HSPC-derived subclones shared the same cellular lineage and evolution pathways, their phylogenetic trees were reconstructed. AML clonal evolution from diagnosis to relapse was investigated in HSPCs derived from three patients (LCL440, LCL465, LCL230), using phylogenetic lineage reconstruction.

These patients were categorized as “intermediate–high risk” according to the European LeukemiaNet classification [21]. They were uniformly treated with the “7+3” chemotherapy protocol, and none underwent allogeneic stem cell transplantation in first complete remission (Table S1).

**Figure 3:**
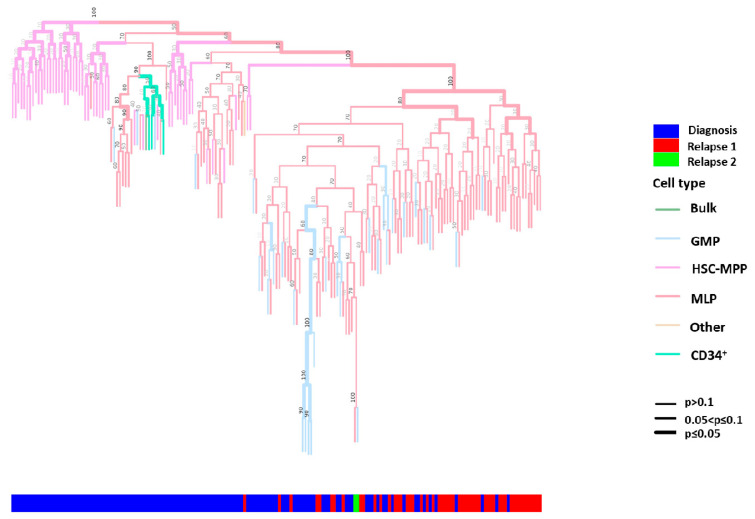
The phylogenetic tree of HSPCs derived from patient LCL440 at diagnosis and relapse. Each tip represents a single cell or a single-cell-derived subclone. Colors in the tree represent different HSPC subpopulations: green—bulk cells; light blue—GMP; light hot pink—HSC-MPP; orchid pink—MLP; light brown—other: hCD45^+^ cells derived from subclones post-injection to mice; light green—CD34^+^. The root is calculated from an average signal of all HSC-MPP samples. The width of the colored branches represents the level of clustering significance (wider = lower *p*-value). The color bar below demonstrates cell populations derived at: blue—diagnosis, red—first relapse, green—second relapse. The *p*-value is calculated using a hypergeometric test. HSPC: hematopoietic stem and progenitor cell; HSC: hematopoietic stem cell; MPP: multipotent progenitor; MLP: multipotent lymphoid progenitor; GMP: granulocyte–monocyte progenitor.

The cell lineage tree of patient LCL440 exhibited distinct clusters at diagnosis and relapse. The HSPC distribution at diagnosis was significantly less dense than at relapse (*p* < 0.05; Figure 3). The relapse cluster was found to originate from both MLP-like and GMP-like subpopulations, already present at diagnosis, suggesting clonal selection/evolution of these subpopulations.

**Figure 4:**
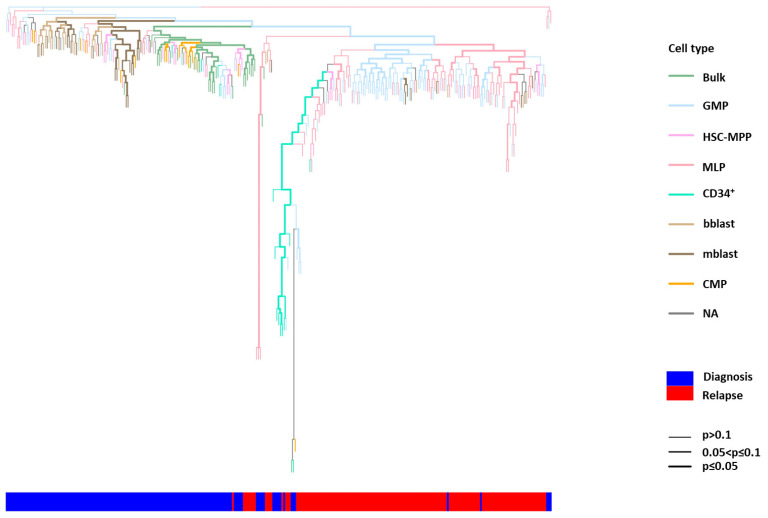
The phylogenetic tree of HSPCs derived from patient LCL465 at diagnosis and relapse. Each tip represents a single cell or a single-cell-derived subclone. Colors in the tree represent different HSPC subpopulations: green —bulk cells; light blue—GMP; pink lace—HSC-MPP; orchid pink—MLP; light green—CD34^+^; beige—bblasts; light brown—mblasts; yellow—CMP; grey—NA. The root is calculated from an average signal of all HSC-MPP samples. The width of the colored branches represents the level of clustering significance (wider = lower *p*-value). The color bar below demonstrates cell populations derived at the following: blue—diagnosis, red—relapse. The *p*-value is calculated using a hypergeometric test. HSC: hematopoietic stem cell; MPP: multipotent progenitor; CMP: common myeloid progenitor; MLP: multipotent lymphoid progenitor; GMP: granulocyte–monocyte progenitor.

Similarly, in patients LCL465 [mixed-phenotype acute leukemia (MPAL)] and LCL230, a clear separation between the diagnosis and relapse clusters was demonstrated (*p* < 0.05), and the relapse cluster was composed of MLP-like and GMP-like HSPC subpopulations, already present at diagnosis. At diagnosis, the depth of HSPC location in the phylogenetic tree was significantly reduced compared to that observed at relapse (*p* < 0.05; Figure 4 and Figure S2).

### 2.4. Leukemogenic Potential of Various HSPC Subpopulations In Vivo

**Figure 5:**
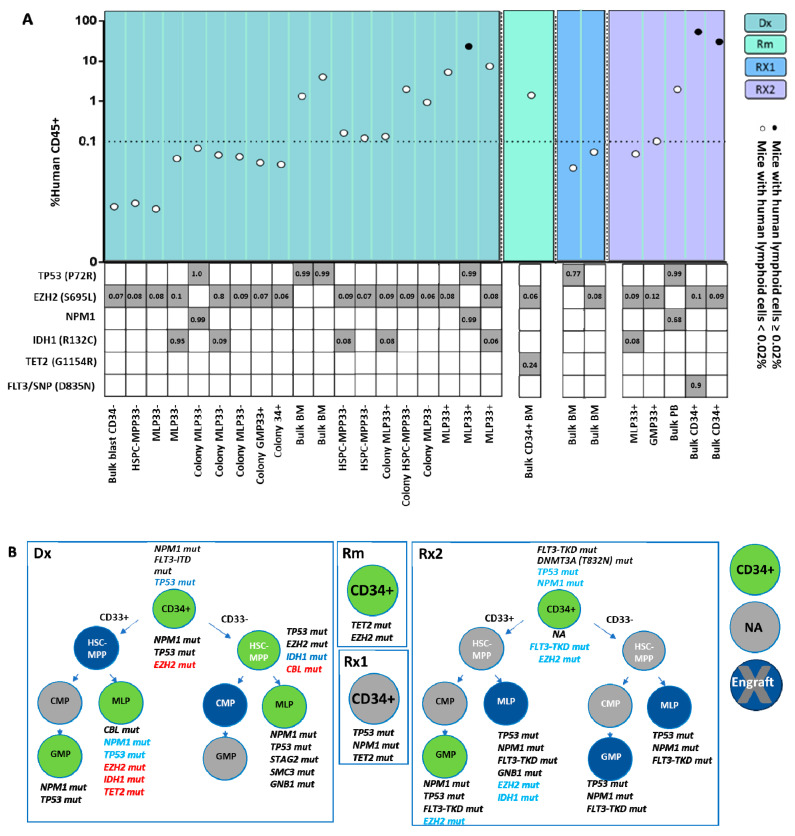
Evaluation of the leukemogenic potential in HSPCs derived from LCL440 patient. (**A**) Bulk cells, HSPCs, or single-HSPC-derived colonies (obtained using the CFU assay) were collected from sacrificed mice and analyzed for the presence of the human CD45^+^ (hCD45^+^) cell marker. The cells that repopulated in vivo for more than 8 weeks in primary transplantation were considered leukemic HSPCs. The percentage of hCD45^+^ cells out of the total number of mouse-derived cells is demonstrated. The dotted line indicates the generally accepted engraftment threshold set at 0.01. In this study focusing on single HSPCs and not on bulk cells, the threshold for engraftment detection was set at 0.02% of human CD45^+^ (hCD45^+^) cells. Cell engraftment of ≥5% is represented as black circles; cell engraftment of <5% is represented as white circles. The percentage of variant allelic frequency (VAF) of detected lesions in sorted hCD45^+^ cells post-transplantation is indicated in the table depicting co-mutations. (**B**) HSPC clonal composition at diagnosis, remission, and relapse. Green circles represent successful engraftment. Grey circles represent HSPCs unavailable for transplantation. Blue circles represent non-overt leukemic cell engraftment. AML-associated detected mutations are specified near each circle and colored according to their detection origin: black for mutations detected only in the bulk cells before injection; blue for mutations detected only after injection to mice; red for mutations detected only in single cells (colonies) before or after injection to mice. Dx: diagnosis; Rm: remission; Rx: relapse; HSC: hematopoietic stem cell; MPP: multipotent progenitor; CMP: common myeloid progenitor; MLP: multipotent lymphoid progenitor; GMP: granulocyte–monocyte progenitor; BM: bone marrow; PB: peripheral blood.

**Figure 6:**
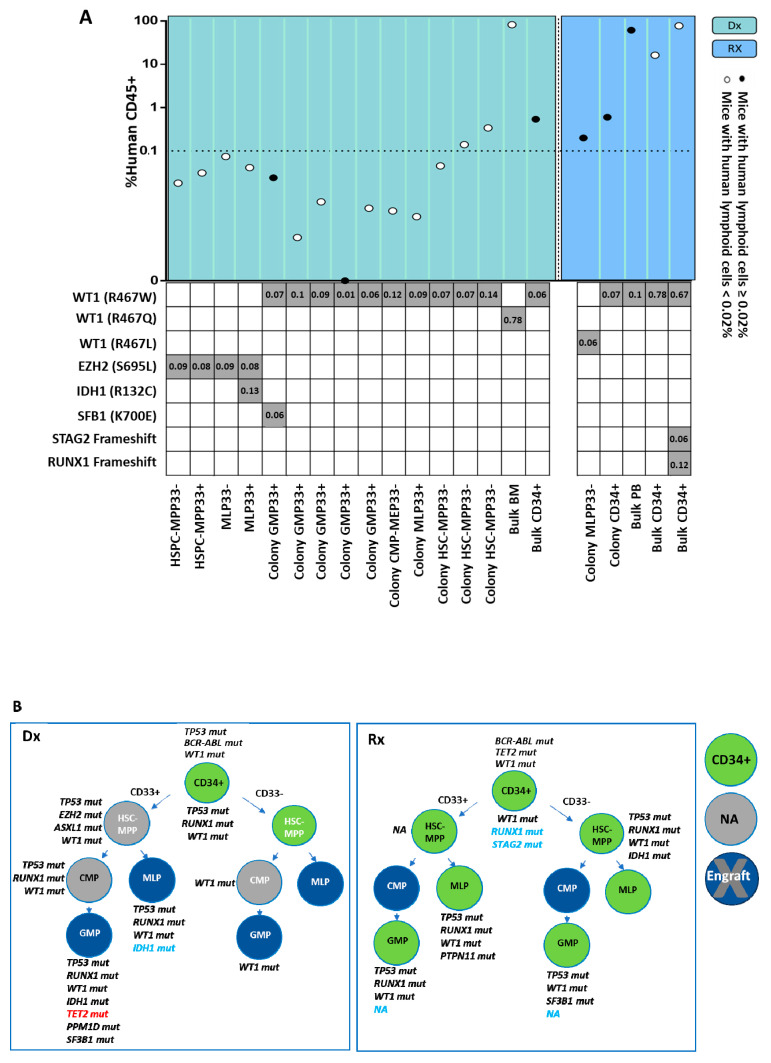
Evaluation of the leukemogenic potential in HSPCs derived from LCL465 patient. (**A**) Bulk cells, HSPCs, or single-HSPC-derived colonies collected from sacrificed mice were analyzed for the presence of the hCD45^+^ cell marker. The percentage of hCD45^+^ cells out of the total number of mouse-derived cells is demonstrated. The dotted line indicates the generally accepted engraftment threshold set at 0.01. In this study focusing on single HSPCs and not on bulk cells, the threshold for engraftment detection was set at 0.02% of human CD45^+^ (hCD45^+^) cells. Cell engraftment of ≥5% is represented as black circles; cell engraftment of <5% is represented as white circles. The percentage of VAF of detected lesions in sorted hCD45+ cells post-transplantation is indicated in the table depicting co-mutations. (**B**) HSPC clonal composition at diagnosis and relapse. Green circles represent successful engraftment. Grey circles represent HSPCs unavailable for transplantation. Blue circles represent non-overt leukemic cell engraftment. AML-associated detected mutations are specified near each circle and colored according to their detection origin: black for mutations detected only in the bulk cells before injection; blue for mutations detected only after injection to mice; red for mutations detected only in single cells (colonies) before or after injection to mice. Dx: diagnosis; Rx: relapse; HSC: hematopoietic stem cell; MPP: multipotent progenitor; CMP: common myeloid progenitor; MLP: multipotent lymphoid progenitor; GMP: granulocyte–monocyte progenitor; MEP: megakaryocyte–erythrocyte progenitor; BM: bone marrow; PB: peripheral blood.

As different abnormalities in the genetic composition lead to diverse functional leukemogenic potentials, we analyzed the unique mutational profile and repopulation capacities of HSPCs in 165 xenotransplant NSG mice injected with single HSPCs or single-HSPC-derived subclones, obtained from the three above-mentioned patients at diagnosis and relapse (Figure 5A,B, Figure 6A,B and Figure S3A,B). Engraftment rates amounted to 38%, 42%, and 28% in mice transplanted with cells derived from patients LCL440, LCL465, and LCL563, respectively. Importantly, in some experiments the engraftment rate was below the generally accepted threshold, as specified in the Section 4; however, these cells originated from the human source, survived in animals for a long period, and harbored leukemia-associated mutations. Hence, these results were included in the analysis. Notably, both mutational analyses and xenograft assays demonstrated that engrafted mice developed leukemia, irrespective of the CD33 expression on the transplanted human cells (Figure 5, Figure 6, and Figure S3).

To explore a potential association between genetic profiles of engrafted HSPCs and their ability to regenerate leukemia in NSG mice, these cells were first evaluated for the presence of hCD45^+^ and subsequently subjected to genetic analysis for the detection of leukemic and pre-leukemic mutations. The engraftment was additionally confirmed by the assessment of short tandem repeats (STR) of collected hCD45^+^ cells relative to the patient’s STR-specific signature (Figure S4A–C).

### 2.5. Mutational Profile Analysis of Single-HSPC-Derived Subclones Demonstrates the Presence of a Relapse-Associated Clone at Diagnosis

To identify early putative leukemia-initiating stem cells, we analyzed the clonal composition of the HSPCs derived from the same three AML patients (LCL440, LCL465, and LCL563). HSPCs obtained from patient LCL440 at diagnosis displayed *FLT3-ITD* (allelic ratio 0.7) and *NPM1* mutations in bulk cells. Post-engraftment, sorted hCD45^+^ cells derived at diagnosis exhibited three major AML mutational compositions: *EZH2*, *EZH2+IDH1*, and *TP53+NPM1* (Figure 5A,B). Although the transplantation of a single MLP-like cell-derived subclone provided a low percentage of hCD45^+^ cells (0.04–0.07%), the analysis showed the mutational composition identical to that found in the bulk MLP-like cells. These findings indicated the heterogeneity of this subpopulation at diagnosis, which included several clones capable of leukemia induction in xenografts. Notably, this patient experienced two relapses, in which the three aforementioned major mutational compositions detected at diagnosis reappeared during relapses. However, at the second relapse, an additional mutation was detected in *FLT3-TKD*, which, along with *EZH2*, propagated overt leukemia. Therefore, in this patient, *EZH2* was an early event associated with leukemia relapse. Importantly, among the HSC-MPP33^−^-like, GMP33^+^-like, and MLP33^+/−^-like HSPC subpopulations identified at diagnosis and capable of generating overt leukemia, only GMP33^+^-like cells were abundant at the second relapse (Figure 2A,B and Figure 3). Notably, the latter subpopulation was enriched following daunorubicin treatment. However, while this fraction was eliminated following the exposure to Ara-C, the MLP33^+^-like cells were not entirely eradicated (Figure S1B). In this patient, the second relapse was associated with the MLP33^+/−^-like subpopulation containing a chemo-resistant clone harboring *TP53+NPM1* mutations. *EZH2* was identified in the majority of engrafted cells obtained from diagnosis and relapse.

The hCD45^+^ cells derived from patient LCL465 with MPAL (Philadelphia chromosome) at diagnosis exhibited two major mutational compositions: *EZH2^+/−^ IDH1* and *WT1^+/−^ SF3B1* post-engraftment (Figure 6A). The HSPC subpopulation capable of generating leukemia at diagnosis was found to be HSC-MPP33^−^-like, while the corresponding subpopulations at relapse were as follows: HSC-MPP33-like, harboring *WT1*, *RUNX1*, and *STAG2* mutations; MLP33^+^-like carrying *TP53*, *WT1*, *IDH1*, and *RUNX1* mutations as well as GMP33^+^-like exhibiting *TP53*, *WT1*, *IDH1*, *RUNX1*, and *TET2* mutations (Figure 2A,C and Figure 6B). Moreover, SCA enabled the identification of low-frequency mutations potentially causing relapse. *TET2* was detected in two single-HSPC-derived subclones at diagnosis and in the bulk DNA at relapse. *STAG2*, present in single-HSPC-derived subclones at diagnosis, was also revealed in CD34^+^ HSPCs obtained at relapse, which became evident only upon engraftment. Both at diagnosis and relapse, *IDH1* appeared only in HSPCs, and not in bulk cells. Importantly, although *WT1* was present in all leukemia-generating clones and in bulk cells derived at diagnosis, an association with other mutations (specifically, *RUNX1* or *TP53*) was required for it to exert the full-blown leukemic potential (Figure 6A,B). In ex vivo experiments, GMP33^+^-like and MLP33^+^-like HSPC subpopulations were found to be resistant to Ara-C, whereas GMP33^+^-like cells were resistant to daunorubicin (Figure S1C). Despite the chemotherapy pressure, the HSC-MPP33^−^-like subpopulation was resistant to both agents.

Post-engraftment, bulk cells and the MLP-like subpopulation obtained from patient LCL563 (del 17p) at diagnosis were found to carry *EZH2* and *EZH2^+/−^ IDH* mutations, respectively. Additionally, a combination of *EZH2* and *NRAS* mutations was identified in the HSC-MPP33^−^-like subpopulation originating from samples obtained at diagnosis. In the vast majority of engrafted single HSPCs and their subpopulations obtained at relapse, *EZH2* was detected. Furthermore, a combination of *EZH2* and *NRAS* was observed in GMP33^−^-like and GMP33^+^-like subpopulations and in bulk cells, derived from samples obtained from relapsed patients (Figure 2A and Figure S3). The fact that the *EZH2* mutation appeared in all engrafted HSPCs, either alone or in combination with other mutations evolving during the disease course, could imply its role as a driver mutation.

Overall, these findings demonstrate the leukemogenic potential of single HSPCs and their contribution to AML relapse.

## 3. Discussion

This research advances the current knowledge by exploring AML relapse mechanisms at the resolution of HSPC subpopulations and single-HSPC-derived subclones. The identification of relapse-initiating HSPCs at diagnosis is crucial and may prevent post-treatment disease progression. To investigate the dynamics of leukemic HSPC subpopulations during AML development, their genetic composition and leukemogenic potential at diagnosis and relapse were evaluated in samples derived from 15 patients, who expressed high levels of CD34^+^/CD38^−^ in the blast fraction, as these primitive leukemic stem cells are considered markers for poor prognosis due to high relapse risk [10,22]. Experiments using HSPCs obtained from primary cells are known to be highly challenging [23]. Hence, according to the study design and based on previous similar studies [24,25], further combined in-depth analyses, including the construction of phylogenetic lineage trees, in vivo leukemogenic assays, and genetic profiling, were performed in samples obtained from 3 of the 15 patients.

SCA has its inherent shortcomings associated with the in vitro culturing of primary AML cells. The major deficiency is related to an insufficient amount of primary cell-derived material, which restricts multiple evaluations [26]. To overcome these pitfalls, we designed a unique medium that preserves the HSPC stemness and ensured the successful generation of single-cell-derived subclones from each leukemic HSPC subpopulation. These cell fractions demonstrated abnormal distribution already in MDS, which became more pronounced at AML diagnosis and relapse. The dominance of MLP33^+/−^-like subpopulations in both disease states was somewhat unexpected, particularly the finding that these subpopulations appeared to be the key ones at relapse. Previous reports demonstrated the presence of MLP-like progenitors in CD34^+^ cells derived from AML patients at diagnosis and the ability of these cells to induce leukemia [11]. In the study by Shlush et al., MLP clones were found to carry the genetic signature of major clones represented in relapse [27], which emphasized their involvement in disease progression. The present study expanded these findings to the pre-leukemic state, showing an association of this cell fraction with clonal evolution leading to relapse. The aberrant HSPC distribution also reflected the skewing of the phenotypic hierarchy, observed in normal hematopoiesis [13]. Moreover, this raised the question of whether a strict hematopoietic hierarchy was maintained in AML or whether the HSPC distribution became completely chaotic.

In this study, the reconstruction of lineage trees for HSPCs obtained at diagnosis and relapse enabled the precise identification of clones and mechanisms driving relapse. The calculation of the relative genetic distance between HSPCs showed that the relapse cluster emerged from either a combination of MLP-like and GMP-like subpopulations or from the MLP-like subpopulation alone, which supported the hypothesis of a preserved hematopoietic hierarchical paradigm in leukemogenesis.

Possible relapse mechanisms suggested in earlier studies were chemo-resistance of hematopoietic stem cells secondary to their quiescent state [28,29,30] and genetic composition that could be either present at diagnosis or acquired during disease evolution [4,31,32]. The contribution of molecular and genetic profiles of leukemic cells to chemo-resistance mechanisms has previously been studied ex vivo at the level of AML CD34^+^ mononuclear cells [4,23,30]. In the present study, ex vivo exposure of the GMP33^+^-like subpopulation to chemotherapeutic agents failed to eradicate it. The MLP33^+^-like subpopulation, despite being largely eradicated after exposure to the same drugs, re-induced leukemia, as demonstrated in engrafted mice. Hence, this subpopulation may represent leukemic HSPCs possibly due to harboring either newly acquired or persistent leukemogenic mutations. While being in line with our previously reported data [33], the current findings further advance the understanding of the heterogeneity of relapse-initiating clones and the complexity of the involved mechanisms at the level of HSPC subpopulations.

To define the leukemogenic potential, i.e., the functionality of HSPCs responsible for relapse, engraftment of these cells in mice is mandatory. While generally, the presence of ≥0.1% of hCD45^+^ cells is considered an acceptable engraftment threshold, by using single HSPCs and HSPC-derived subclones, we managed to detect the human cell engraftment with a much lower threshold of ≥0.02%, thus ensuring significantly higher sensitivity. Furthermore, the fact that leukemia engraftment was observed in mice regardless of the CD33 expression in the injected human cells may question the specificity of this antigen as a leukemic marker. Hence, we suggest that the detection of the MLP/GMP HSPC subpopulations in patient samples at diagnosis could point to the risk of AML relapse.

Subsequent genetic SCA of engrafted single HSPCs and HSPC-derived subclones using next-generation sequencing (NGS) with a panel of common AML mutations demonstrated that relapse-associated subpopulations were endowed with a specific genetic makeup, i.e., mutated *EZH2* and *TP53,* which were present already at diagnosis. Notably, while *EZH2* mutation is reported in 4% of AML cases [34], in this study, all the patients exhibited *EZH2* (*S695L*) in exon 18, which has not been commonly described in AML. The importance of *EZH2* in leukemogenesis was emphasized by the finding that 54% of engrafted mice harbored this mutation, with a high probability of co-mutation between *EZH2* and *IDH1/NRAS*. Similarly, Stasik et al. demonstrated co-occurrence of *EZH2* with *NRAS* mutations in 25% of AML patients [34]. Yet, we found that *EZH2* was capable of generating leukemia in mice even in the absence of *IDH1* or *NRAS*. The presence of the specific *EZH2* point mutation (*S695L*) in the patients analyzed in-depth in the current study does not seem to be an accidental finding and could be detected only using SCA at the resolution of HSPCs. Moreover, the majority of clones with mutated *EZH2* were indeed chemo-resistant, which was consistent with previously reported data [35]. Our findings that the cells harboring the *EZH2* mutation exhibited long-term survival after transplantation to mice (over 29 weeks) or after exposure to chemotherapy agents could suggest that this mutation might adapt to the changing environment and occur as an early event.

*TP53* is one of the most well-studied tumor suppressor genes. Its mutations are associated with poor prognosis and decreased response to chemotherapy in AML patients [24,36]. The current study demonstrated the presence of *TP53* (*P72R*) in the majority of HSPCs obtained at diagnosis, and its reappearance in relapse clones. These findings may have important clinical implications, as pharmacological targeting of the *TP53* pathway may provide a novel therapeutic option in AML. Several clinical trials aiming to inhibit *TP53*-mutated cells (arsenic trioxide) or reactivate *TP53* (APR-246) are currently ongoing.

The major strength of this study is the novel observation that in all three patients analyzed in detail, the predominant leukemic HSPC subpopulations were MLP and GMP, particularly in relapsed disease (based on the phylogenetic reconstruction). Although only three patients were analyzed, these results are still remarkable and may have broader implications. While previous studies have shown that MLP/GMP cell subpopulations can propagate AML, their predominance both at diagnosis and, even more so, at relapse, demonstrated in our research, is meaningful and may inspire further studies to confirm and extend these data in larger patient cohorts. This study has limitations. While all the experiments have been conducted using HSPCs obtained from primary cells, this is challenging and explains the small number of patients included in the combined analyses.

In conclusion, this study employed combined functional and genomic analyses to evaluate ex vivo and in vivo the leukemogenic potential and mutational profile of primary AML HSPCs. The revealed AML oncogenic makeup was characterized by the abundant presence of MLP-like or GMP-like subpopulations carrying mutated *EZH2* and *TP53* both at diagnosis and relapse. To the best of our knowledge, this is the first study to demonstrate, using the unique culture medium, SCA, and phylogenetic reconstruction, that specific AML HSPC subclones and single HSPCs share the same lineage and evolution pathways, which collectively points to their role in leveraging disease propagation.

## 4. Materials and Methods

### 4.1. Human Specimens

Matched diagnosis and relapse BM samples were collected from 15 AML patients (Table S1) and preserved at the Rambam Biobank. BM samples of 6 MDS patients and those derived from 7 age-matched HDs served as control. HD specimens were obtained from individuals who underwent elective hip replacement surgery.

### 4.2. HSPC Isolation and Culture

This study focused on AML patients with high CD34^+^ expression (>60%) in the blast fraction. CD34^+^ cells were isolated from the BM (following mononuclear cell separation) of AML patients by positive selection using anti-human CD34 microbeads (Miltenyi Biotec, Bergisch Gladbach, Germany). Enriched CD34^+^ cells were fractionated into leukemic (CD33^+^) and non-leukemic (CD33^−^) HSPCs. They were further sorted using FACSariaII (BD Bioscience, San Jose, CA, USA) into the following HSPC subpopulations (Figure 1A) according to immunophenotypic profiles [25]: HSC-MPP, GMP, CMP-MEP, and MLP (Table S2). The sorted fractions were used in the colony-forming unit (CFU) assay or the xenotransplantation assay. The CFU assay was adjusted for the generation of single-cell-derived subclones. All experiments were performed with mycoplasma-free cells.

### 4.3. Generation of Single-Cell-Derived Subclones from Sorted HSPCs

SCA using a modified technique capable of tracking single-HSPC-derived subclones during AML evolution was employed. To overcome SCA restrictions—mainly related to a limited number of cells available for required evaluations and the rarity of HSPCs in the BM—we generated a setup of single-cell-derived subclones that preserved the stemness of every leukemic HSPC subpopulation. Each subclone was evaluated for chemo-resistance, in vivo leukemogenic potential, and mutational profile. The subclone cell of origin was identified using phylogenetic tree reconstruction.

Sorted HSPCs obtained from AML patient samples at diagnosis and relapse were cultured in a unique medium designed in the current study to produce single-cell-derived subclones from each HSPC subpopulation. This medium included 1% standard methylcellulose medium (StemCell Technologies, Vancouver, BC, Canada, # H4100) supplemented with 37% FBS, 1.2% bovine serum albumin (BSA), 1% L-glutamine, 10^−6^ M β-mercaptoethanol, 1 IU/mL of human erythropoietin (EPO) (PeproTech, Cranbury, NJ, USA, #100-64), 50 ng/mL of human stem cell factor (SCF) (PeproTech #300-07), 25 ng/mL of human FLT3 ligand (GenScript, Piscataway, NJ, USA, #Z02923), 10 ng/mL of interleukin 3 (IL-3) (GenScript #Z03156), 10 ng/mL of interleukin 6 (IL-6) (GenScript #Z03034), 10 ng/mL of granulocyte colony stimulating factor (G-CSF) (PeproTech, #300-23), 5 ng/mL of granulocyte–macrophage colony-stimulating factor (GM-CSF) (PeproTech, #300-03), 10 ng/mL of human thrombopoietin (TPO) (PeproTech, #300-18), 100 IU mL penicillin, and 100 mg/mL streptomycin. Colonies were counted after 14 days; each of them was collected and split for either whole genome amplification and genotyping assays, or for immediate transplantation to NSG mice to conduct leukemogenic potential assays.

### 4.4. In Vivo Leukemogenic Potential Assays

NOD.Cg-PrkdcscidIl2rgtm1Wjl/SzJ (NSG) female mice (Jackson Laboratory, Bar Harbor, ME, USA) were bred under specific pathogen-free conditions. Twenty-four hours pre-transplantation, 8–12-week-old female NSG mice were sub-lethally irradiated with 225 cGy. Primary human cells were intra-femorally injected into mice. Since human hematopoietic stem cells are capable of long-term multi-lineage engraftment [13,15], animals were sacrificed 8–30 weeks post-transplantation or if tumors developed in the injected femur. BM was then harvested and the engraftment was evaluated by flow cytometry using the following anti-human antibodies: CD45, CD33, CD34, CD38, CD15, and CD19. Cell viability was assessed with propidium iodide (BioLegend, San Diego, CA, USA). Analyses were performed using FlowJo software v.10 (Treestar Inc., Ashland, OR, USA), and the marker expression was presented as the percentage of stained cells.

All flow cytometry analyses were done using an LSR II or FACSariaII (BD Bioscience) device. Engraftment of >5% of hCD45^+^ cells with the major CD33^+^ population (>60%) was classified as overt leukemia; the presence of 0.1–5% of hCD45^+^ cells with <60% of the CD33^+^ population was classified as non-overt leukemia; the presence of <0.1% of hCD45^+^ population was considered non-engraftment [13,14]. However, in this study, focusing on single HSPCs, the engraftment threshold was set at 0.02% of hCD45^+^ cells (unlike the generally accepted threshold of 0.1% for bulk cells).

### 4.5. Lineage Tree Reconstruction

Phylogenetic lineage was reconstructed by tracking random somatic mutations in non-coding regions of DNA [specifically, microsatellites, known as STR]. The lineage reconstruction of single HSPCs and single-HSPC-derived subclones was done based on the strategy developed by the Shapiro group for high throughput Illumina-NGS-compatible, STR-targeting molecular inversion probes (MIPs).

Whole genome amplification (prerequisite): Genomic DNA from single cells and bulk samples was subjected to whole genome amplification (WGA), using the commercially available Ampli1^TM^ WGA Kit (Ampli1^TM^ WGA, Menarini Silicon Biosystems, Bologna, Italy).

Phylogenetic trees were reconstructed using the duplex-MIP protocol [37], supported by the laboratory information management system (LIMS). This step was followed by STR-dedicated analysis. The key steps, including targeted enrichment, mapping, genotyping, phylogenetic reconstruction, bootstrap validation, and visualization, are briefly summarized below.

Targeted enrichment: Duplex molecular inversion probes (MIPs, alternatively known as padlock probes) were derived from precursors that were synthesized on a high-resolution microarray. A panel of duplex MIPs targeting highly mutable STRs are hybridized to single-cell whole genome amplified genomic DNA (gap-filled, ligated). Standard Illumina libraries were constructed using PCR with unique barcoded primers; then, NGS was applied, targeting ~1 M reads/sample.

Mapping: Merged R1 and R2 reads were mapped against an index of STR variations (consisting of all targeted panel amplicons) according to their sequence in the reference genome. Additionally, these reads were repeated with variations in the number of repeat units in the STR parts of the amplicon.

Genotyping: In vitro processing of genomic STRs resulted in typical stutter patterns. Hence, model stutter patterns were calibrated using a synthetic library spanning that included all naturally occurring genotypes of mono and di-repeat STRs. The sample-derived stutters were then genotyped with the R&B method [38] using a KDtree search among all possible model stutter patterns.

Lineage tree reconstruction: The tree reconstruction was performed using the Fasttree 2 algorithm [39]. Genotyped STR repeat numbers were arbitrarily translated to characters, concatenated by a fixed panel order to a sequence-like form, and fed as fasta input into FastTree.

Bootstrapping: The reconstructed tree was validated by repeating the reconstruction heuristic 1000 times with perturbations. In each iteration, the loci were sampled with replacement so that the same number of loci was fed into the reconstruction, but some loci appeared more than once, while others had not been sampled at all. On average, 30~40% of the data were dropped in every iteration. The resulting trees were aggregated using Booster [40] to provide transfer bootstrap expectation (TBE) confidence values for every internal node.

Visualization: The reconstructed lineage tree (with or without bootstrap values), stored as a standard NEWICK file, could be depicted using any dedicated tool. Specifically, we developed the LineagePlotter platform, which allowed to color the branches of the tree according to predefined classifications and to display the hypergeometric *p*-value score of the branches as the width of each branch [41]. These *p*-values signified the probability of observing an equal or greater number of leaves with a given color annotation, assuming a random sampling process from the pool of all the leaves. Bootstrap values could also be displayed over the tree's internal nodes.

### 4.6. MIP Analysis for Mutational Profile Determination

A DNA template (1 µL) was added to a hybridization mix together with an MIP pool (final concentration of 0.05 pM per probe) in 1x Ampligase buffer (Epicentre Biotechnologies, Madison, WI, USA). The mix was incubated in a thermal cycler: 98 °C for 3 min, 85 °C for 30 min, 60 °C for 60 min, and 56 °C for 1 or 2 overnight incubations. The product was mixed with dNTPs (Larova, Jena, Germany; 15 pM), Betaine (375 mM, Sigma-Aldrich, Saint Louis, MO, USA), NAD^+^ (1 mM, New England Biolabs (NEB), Ipswich, MA, USA), additional Ampligase buffer (0.5x), Ampligase (total of 1.25U, Epicentre, Paris, France), and Phusion HF (0.16U, NEB). The mix was incubated at 56 °C for 60 min, followed by 72 °C for 20 min. Enzymatic digestion of linear probes was performed by adding Exonuclease I (4U, NEB) and Exonuclease III (25U, NEB), with consequent incubation at 37 °C for 2 h, followed by 80 °C for 20 min. The final product was amplified using iProof HF Master Mix (Bio-Rad Laboratories, Hercules, CA, USA). Samples were pooled and concentrated using AMPure XP beads (Beckman Coulter, Brea, CA, USA) at a 1.3x volumetric concentration, size-selected (190–370 bp) using Blue Pippin (Sage Science, Beverly, MA, USA), and sequenced in either NextSeq or Novaseq6000 (Illumina, San Diego, CA, USA) 2x151bp paired-end runs using custom primers as described in the past [42] (Table S3).

### 4.7. Targeted MIP Panel Sequencing Variant Calling

Paired-end 2x151bp sequencing data were converted to FASTQ format. Reads were merged using BBmerge v38.62 [43] with default parameters, followed by trimming of the ligation and extension arms using Cutadapt v2.10 [44]. UMIs were trimmed and assigned to each read header. Processed reads were aligned using BWA-MEM to a custom reference genome, comprised of the MIP ARCH panel sequences ±150 bases extracted from broad HG19 [45]. Aligned files were sorted and converted to BAM (SAMTools V1.9 [46]), followed by Indel realignment using AddOrReplaceReadGroups (Picard tools) and later IndelRealigner [GATK v.3.7 [47]]. Variant calling was done using mpileup for the single nucleotide variant and Varscan2 v2.3.9 [48] and Platypus v0.8.1 [49] for indels. Variants were annotated using ANNOVAR subversion 322 [50].

### 4.8. Chimerism Analysis

The AmpFlSTR NGM SElect Express PCR Amplification Kit (ThermoFisher, Waltham, MA, USA) was used to amplify polymorphic STR markers. The amplified loci were further assessed using the ABI PRISM 3500 Genetic Analyzer (Applied Biosystems, Foster City, CA, USA); peak areas were estimated with the Gene Mapper software v6.1 (Applied Biosystems). The assay detection limit was about 1–2%, with a quantitation limit of 5%.

### 4.9. Ex Vivo Chemo-Resistance Assay

Enriched CD34^+^ primary AML cells were co-cultured with the MS-5 cell line (mouse bone marrow) (RRID: CVCL_2128; DSMZ-German Collection of Microorganisms and Cell Cultures, Braunschweig, Germany, #ACC 441) as a feeder layer (passages 6–13) in the MyeloCult H5100 media (StemCell Technologies, #05100), supplemented with 1% PS; 100 ng/mL SCF; 50 ng/mL TPO; 20 ng/mL interleukin-7 (IL-7) (PeproTech, #200-07); 10 ng/mL IL-3; 20 ng/mL IL-6; 10 ng/mL Flt-3 Ligand (Flt-3L); 20 ng/mL G-CSF; and 20 ng/mL GM-CSF. Following 72 h of co-culture, chemotherapy agents Ara-C and daunorubicin (Sigma-Aldrich, #C1768 and #30450, respectively) at concentrations of 10,000–20,000 nM and 250–500 nM, respectively, were applied to the cells for additional incubation (48–72 h). These doses were selected following dose-response calibration. CD34^+^ cells were then stained with Annexin-V/PI and their survival was analyzed with FACSariaII. The chemo-resistance threshold was set at 60% cell viability. The HSPC subpopulation ratio was evaluated pre- and post-exposure using the antibody panel (Table S2). FlowJo V10 software was employed for analyses.

## Data Availability

The sequencing datasets generated during this study are available at the ArrayExpress data repository (https://www.ebi.ac.uk/arrayexpress/experiments/E-MTAB-11884), accession number E-MTAB-11884, accessed on 12 August 2022 and at https://www.ebi.ac.uk/biostudies/arrayexpress/studies/E-MTAB-12238, accession number E-MTAB-12238, accessed on 12 November 2022.

## Supplementary Materials

The supporting information can be downloaded at: https://www.mdpi.com/article/10.3390/ijms26094224/s1.

## References

[1] Papaemmanuil E., Gerstung M., Bullinger L., Gaidzik V.I., Paschka P., Roberts N.D., Potter N.E., Heuser M., Thol F., Bolli N. (2016). Genomic Classification and Prognosis in Acute Myeloid Leukemia. N. Engl. J. Med..

[2] Dohner K., Thiede C., Jahn N., Panina E., Gambietz A., Larson R.A., Prior T.W., Marcucci G., Jones D., Krauter J. (2020). Impact of NPM1/FLT3-ITD genotypes defined by the 2017 European LeukemiaNet in patients with acute myeloid leukemia. Blood.

[3] Fennell K.A., Vassiliadis D., Lam E.Y.N., Martelotto L.G., Balic J.J., Hollizeck S., Weber T.S., Semple T., Wang Q., Miles D.C. (2022). Non-genetic determinants of malignant clonal fitness at single-cell resolution. Nature.

[4] Morita K., Wang F., Jahn K., Hu T., Tanaka T., Sasaki Y., Kuipers J., Loghavi S., Wang S.A., Yan Y. (2020). Clonal evolution of acute myeloid leukemia revealed by high-throughput single-cell genomics. Nat. Commun..

[5] Bullinger L., Dohner K., Dohner H. (2017). Genomics of Acute Myeloid Leukemia Diagnosis and Pathways. J. Clin. Oncol..

[6] Welch J.S., Ley T.J., Link D.C., Miller C.A., Larson D.E., Koboldt D.C., Wartman L.D., Lamprecht T.L., Liu F., Xia J. (2012). The origin and evolution of mutations in acute myeloid leukemia. Cell.

[7] Klco J.M., Spencer D.H., Miller C.A., Griffith M., Lamprecht T.L., O’Laughlin M., Fronick C., Magrini V., Demeter R.T., Fulton R.S. (2014). Functional heterogeneity of genetically defined subclones in acute myeloid leukemia. Cancer Cell.

[8] Schwede M., Jahn K., Kuipers J., Miles L.A., Bowman R.L., Robinson T., Furudate K., Uryu H., Tanaka T., Sasaki Y. (2024). Mutation order in acute myeloid leukemia identifies uncommon patterns of evolution and illuminates phenotypic heterogeneity. Leukemia.

[9] Miles L.A., Bowman R.L., Merlinsky T.R., Csete I.S., Ooi A.T., Durruthy-Durruthy R., Bowman M., Famulare C., Patel M.A., Mendez P. (2020). Single-cell mutation analysis of clonal evolution in myeloid malignancies. Nature.

[10] Karantanos T., Jones R.J. (2019). Acute Myeloid Leukemia Stem Cell Heterogeneity and Its Clinical Relevance. Adv. Exp. Med. Biol..

[11] Goardon N., Marchi E., Atzberger A., Quek L., Schuh A., Soneji S., Woll P., Mead A., Alford K.A., Rout R. (2011). Coexistence of LMPP-like and GMP-like leukemia stem cells in acute myeloid leukemia. Cancer Cell.

[12] Chopra M., Bohlander S.K. (2019). The cell of origin and the leukemia stem cell in acute myeloid leukemia. Genes Chromosomes Cancer.

[13] Laurenti E., Gottgens B. (2018). From haematopoietic stem cells to complex differentiation landscapes. Nature.

[14] Sugimura R., Jha D.K., Han A., Soria-Valles C., da Rocha E.L., Lu Y.F., Goettel J.A., Serrao E., Rowe R.G., Malleshaiah M. (2017). Haematopoietic stem and progenitor cells from human pluripotent stem cells. Nature.

[15] Notta F., Doulatov S., Laurenti E., Poeppl A., Jurisica I., Dick J.E. (2011). Isolation of single human hematopoietic stem cells capable of long-term multilineage engraftment. Science.

[16] Vosberg S., Greif P.A. (2019). Clonal evolution of acute myeloid leukemia from diagnosis to relapse. Genes Chromosomes Cancer.

[17] Potter N., Miraki-Moud F., Ermini L., Titley I., Vijayaraghavan G., Papaemmanuil E., Campbell P., Gribben J., Taussig D., Greaves M. (2019). Single cell analysis of clonal architecture in acute myeloid leukaemia. Leukemia.

[18] Hughes A.E., Magrini V., Demeter R., Miller C.A., Fulton R., Fulton L.L., Eades W.C., Elliott K., Heath S., Westervelt P. (2014). Clonal architecture of secondary acute myeloid leukemia defined by single-cell sequencing. PLoS Genet..

[19] Paguirigan A.L., Smith J., Meshinchi S., Carroll M., Maley C., Radich J.P. (2015). Single-cell genotyping demonstrates complex clonal diversity in acute myeloid leukemia. Sci. Transl. Med..

[20] Shouval R., Shlush L.I., Yehudai-Resheff S., Ali S., Pery N., Shapiro E., Tzukerman M., Rowe J.M., Zuckerman T. (2014). Single cell analysis exposes intratumor heterogeneity and suggests that FLT3-ITD is a late event in leukemogenesis. Exp. Hematol..

[21] Dohner H., Estey E., Grimwade D., Amadori S., Appelbaum F.R., Buchner T., Dombret H., Ebert B.L., Fenaux P., Larson R.A. (2017). Diagnosis and management of AML in adults: 2017 ELN recommendations from an international expert panel. Blood.

[22] Dimitriou M., Mortera-Blanco T., Tobiasson M., Mazzi S., Lehander M., Hogstrand K., Karimi M., Walldin G., Jansson M., Vonlanthen S. (2024). Identification and surveillance of rare relapse-initiating stem cells during complete remission after transplantation. Blood.

[23] Garcia-Garcia A., Klein T., Born G., Hilpert M., Scherberich A., Lengerke C., Skoda R.C., Bourgine P.E., Martin I. (2021). Culturing patient-derived malignant hematopoietic stem cells in engineered and fully humanized 3D niches. Proc. Natl. Acad. Sci. USA.

[24] Woo J., Howard N.P., Storer B.E., Fang M., Yeung C.C., Scott B.L., Deeg H.J. (2017). Mutational analysis in serial marrow samples during azacitidine treatment in patients with post-transplant relapse of acute myeloid leukemia or myelodysplastic syndromes. Haematologica.

[25] van Spronsen M.F., Hanekamp D., Westers T.M., van Gils N., Vermue E., Rutten A., Jansen J.H., Lissenberg-Witte B.I., Smit L., Schuurhuis G.J. (2023). Immunophenotypic aberrant hematopoietic stem cells in myelodysplastic syndromes: A biomarker for leukemic progression. Leukemia.

[26] Lahnemann D., Koster J., Szczurek E., McCarthy D.J., Hicks S.C., Robinson M.D., Vallejos C.A., Campbell K.R., Beerenwinkel N., Mahfouz A. (2020). Eleven grand challenges in single-cell data science. Genome Biol..

[27] Shlush L.I., Mitchell A., Heisler L., Abelson S., Ng S.W.K., Trotman-Grant A., Medeiros J.J.F., Rao-Bhatia A., Jaciw-Zurakowsky I., Marke R. (2017). Tracing the origins of relapse in acute myeloid leukaemia to stem cells. Nature.

[28] Ebinger S., Ozdemir E.Z., Ziegenhain C., Tiedt S., Castro Alves C., Grunert M., Dworzak M., Lutz C., Turati V.A., Enver T. (2016). Characterization of Rare, Dormant, and Therapy-Resistant Cells in Acute Lymphoblastic Leukemia. Cancer Cell.

[29] Behbehani G.K., Samusik N., Bjornson Z.B., Fantl W.J., Medeiros B.C., Nolan G.P. (2015). Mass Cytometric Functional Profiling of Acute Myeloid Leukemia Defines Cell-Cycle and Immunophenotypic Properties That Correlate with Known Responses to Therapy. Cancer Discov..

[30] Malani D., Kumar A., Bruck O., Kontro M., Yadav B., Hellesoy M., Kuusanmaki H., Dufva O., Kankainen M., Eldfors S. (2022). Implementing a Functional Precision Medicine Tumor Board for Acute Myeloid Leukemia. Cancer Discov..

[31] Ding L., Ley T.J., Larson D.E., Miller C.A., Koboldt D.C., Welch J.S., Ritchey J.K., Young M.A., Lamprecht T., McLellan M.D. (2012). Clonal evolution in relapsed acute myeloid leukaemia revealed by whole-genome sequencing. Nature.

[32] Shlush L.I., Zandi S., Mitchell A., Chen W.C., Brandwein J.M., Gupta V., Kennedy J.A., Schimmer A.D., Schuh A.C., Yee K.W. (2014). Identification of pre-leukaemic haematopoietic stem cells in acute leukaemia. Nature.

[33] Shlush L.I., Chapal-Ilani N., Adar R., Pery N., Maruvka Y., Spiro A., Shouval R., Rowe J.M., Tzukerman M., Bercovich D. (2012). Cell lineage analysis of acute leukemia relapse uncovers the role of replication-rate heterogeneity and microsatellite instability. Blood.

[34] Stasik S., Middeke J.M., Kramer M., Rollig C., Kramer A., Scholl S., Hochhaus A., Crysandt M., Brummendorf T.H., Naumann R. (2020). EZH2 mutations and impact on clinical outcome: An analysis in 1,604 patients with newly diagnosed acute myeloid leukemia. Haematologica.

[35] Gollner S., Oellerich T., Agrawal-Singh S., Schenk T., Klein H.U., Rohde C., Pabst C., Sauer T., Lerdrup M., Tavor S. (2017). Loss of the histone methyltransferase EZH2 induces resistance to multiple drugs in acute myeloid leukemia. Nat. Med..

[36] Yan B., Claxton D., Huang S., Qiu Y. (2020). AML chemoresistance: The role of mutant TP53 subclonal expansion and therapy strategy. Exp. Hematol..

[37] Tao L., Raz O., Marx Z., Ghosh M.S., Huber S., Greindl-Junghans J., Biezuner T., Amir S., Milo L., Adar R. (2021). Retrospective cell lineage reconstruction in humans by using short tandem repeats. Cell Rep. Methods.

[38] Raz O., Biezuner T., Spiro A., Amir S., Milo L., Titelman A., Onn A., Chapal-Ilani N., Tao L., Marx T. (2019). Short tandem repeat stutter model inferred from direct measurement of in vitro stutter noise. Nucleic Acids Res..

[39] Price M.N., Dehal P.S., Arkin A.P. (2010). FastTree 2-approximately maximum-likelihood trees for large alignments. PLoS ONE.

[40] Lemoine F., Domelevo Entfellner J.B., Wilkinson E., Correia D., Davila Felipe M., De Oliveira T., Gascuel O. (2018). Renewing Felsenstein’s phylogenetic bootstrap in the era of big data. Nature.

[41] Neumeier Y., Raz O., Tao L., Marx Z., Shapiro E. (2025). Tracking Somatic Mutations for Lineage Reconstruction. Methods Mol. Biol..

[42] Biezuner T., Brilon Y., Arye A.B., Oron B., Kadam A., Danin A., Furer N., Minden M.D., Hwan Kim D.D., Shapira S. (2022). An improved molecular inversion probe based targeted sequencing approach for low variant allele frequency. NAR Genom. Bioinform..

[43] Bushnell B., Rood J., Singer E. (2017). BBMerge-Accurate paired shotgun read merging via overlap. PLoS ONE.

[44] Martin M. (2011). Cutadapt Removes Adapter Sequences from High-Throughput Sequencing Reads. EMBnet J..

[45] Li W., Freudenberg J. (2014). Mappability and read length. Front. Genet..

[46] Li H., Handsaker B., Wysoker A., Fennell T., Ruan J., Homer N., Marth G., Abecasis G., Durbin R., Genome Project Data Processing Subgroup (2009). The Sequence Alignment/Map format and SAMtools. Bioinformatics.

[47] McKenna A., Hanna M., Banks E., Sivachenko A., Cibulskis K., Kernytsky A., Garimella K., Altshuler D., Gabriel S., Daly M. (2010). The Genome Analysis Toolkit: A MapReduce framework for analyzing next-generation DNA sequencing data. Genome Res..

[48] Koboldt D.C., Zhang Q., Larson D.E., Shen D., McLellan M.D., Lin L., Miller C.A., Mardis E.R., Ding L., Wilson R.K. (2012). VarScan 2: Somatic mutation and copy number alteration discovery in cancer by exome sequencing. Genome Res..

[49] Rimmer A., Phan H., Mathieson I., Iqbal Z., Twigg S.R.F., Consortium W.G.S., Wilkie A.O.M., McVean G., Lunter G. (2014). Integrating mapping-, assembly- and haplotype-based approaches for calling variants in clinical sequencing applications. Nat. Genet..

[50] Wang K., Li M., Hakonarson H. (2010). ANNOVAR: Functional annotation of genetic variants from high-throughput sequencing data. Nucleic Acids Res..

