# Supplementary material for: GMP-like and MLP-like Subpopulations of Hematopoietic Stem and Progenitor Cells Harboring Mutated EZH2 and TP53 at Diagnosis Promote Acute Myeloid Leukemia Relapse: Data of Combined Molecular, Functional, and Genomic Single-Stem-Cell Analyses
Source: Int J Mol Sci. 2025 Apr 29;26(9):4224. doi: 10.3390/ijms26094224 (PMC12072498; doi:10.3390/ijms26094224)
Supplement: Supplementary file 1 [file ijms-26-04224-s001.zip › ijms-3561193-supplementary.pdf]

Supporting Information

GMP-like and MLP-like subpopulations of hematopoietic stem and progenitor cells harboring mutated *EZH2* and *TP53* at diagnosis promote acute myeloid leukemia relapse: Data of Combined Molecular, Functional and Genomic Single Stem Cell Analyses

Tal Shahar Gabay, Nofar Stolero, Niv Rabhun, Rawan Sabah, Ofir Raz, Yaara Neumeier, Zipora Marx, Liming Tao, Tamir Biezuner, Shiran Amir, Rivka Adar, Ron Levy, Noa Chapal-Ilani, Natalia Evtiugina, Liran I. Shlush, Ehud Shapiro, Shlomit Yehudai-Resheff*, Tsila Zuckerman*

* These authors contributed equally to this work

Correspondence: s_yehudai@rambam.health.gov.il or t_zuckerman@rmc.gov.il

Contents:

Figures S1-S4

Tables S1-S3

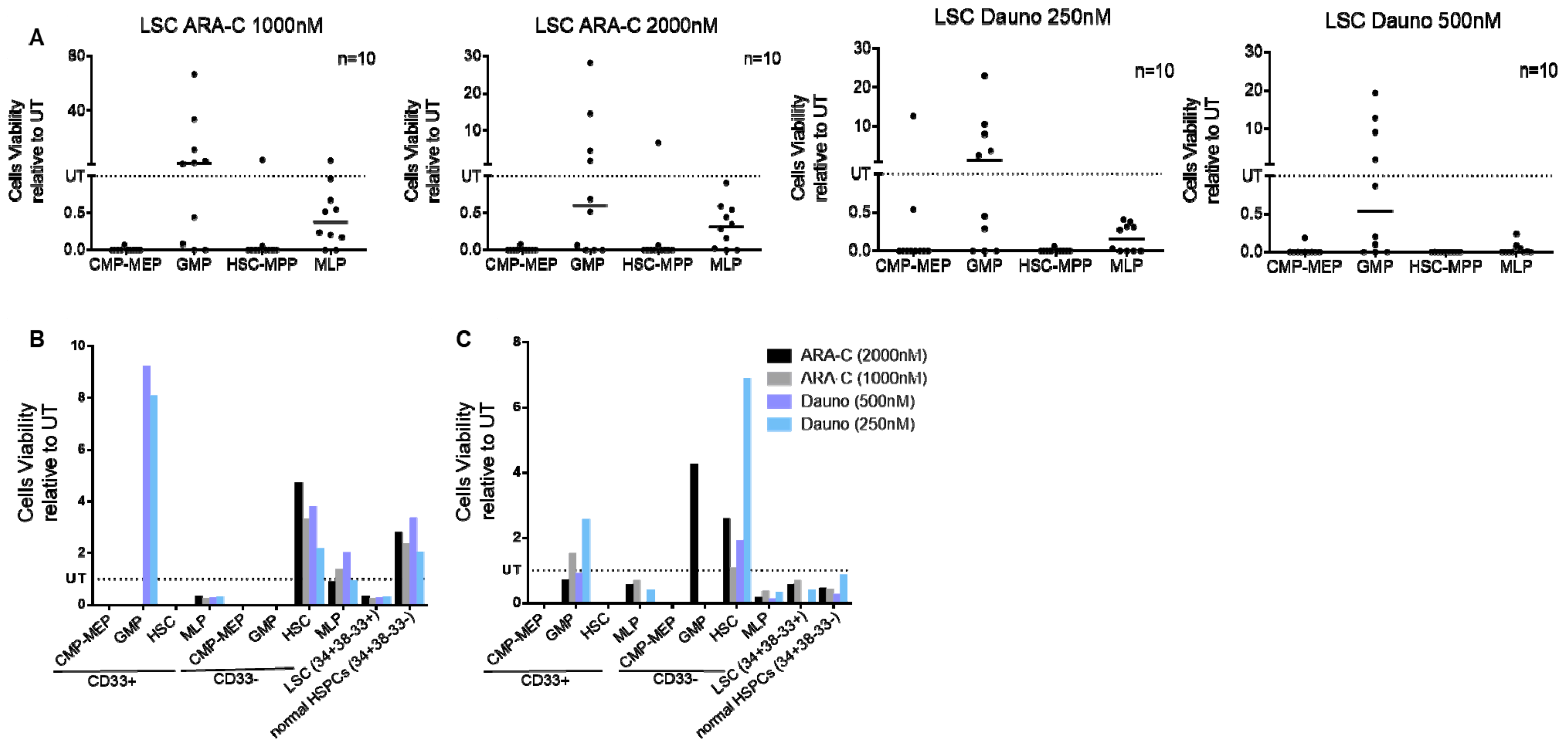
A
LSC ARA-C 1000nM
LSC ARA-C 2000nM
LSC Dauno 250nM
LSC Dauno 500nM
n=10
Cells Viability relative to UT
UT
CMP-MEP
GMP
HSC-MPP
MLP
B
C
ARA-C (2000nM)
ARA-C (1000nM)
Dauno (500nM)
Dauno (250nM)
HSC
CD33+
CD33-
LSC (34+38-33+)
normal HSPCs (34+38-33-)

Figure S1. Viability of leukemic and non-leukemic CD33$^+$ HSPCs derived from AML patients after in vitro exposure to Ara-C (1000 nM and 2000 nM) and daunorubicin (250 nM and 500 nM)

(**A**) Response of leukemic HSPCs, derived from 10 AML patients at diagnosis to in vitro exposure to Ara-C (1000 nM and 2000 nM) and daunorubicin (250 nM and 500 nM). The response was measured as the percentage of viable cells detected post-treatment. Y-axis values present cell viability post-treatment relative to untreated control.
(**B**) Response of leukemic HSPCs, derived from patient LCL440
(**C**) Response of leukemic HSPCs, derived from patient LCL465
Y-axis values present cell viability post-treatment relative to untreated control (dashed line).

HSPC: hematopoietic stem and progenitor cells; HSC: hematopoietic stem cells; MPP: multipotent progenitor; CMP: common myeloid progenitor; MLP: multipotent lymphoid progenitor; GMP: granulocyte-monocyte progenitor; MEP: megakaryocyte-erythrocyte progenitor; Ara-C: cytosine arabinoside

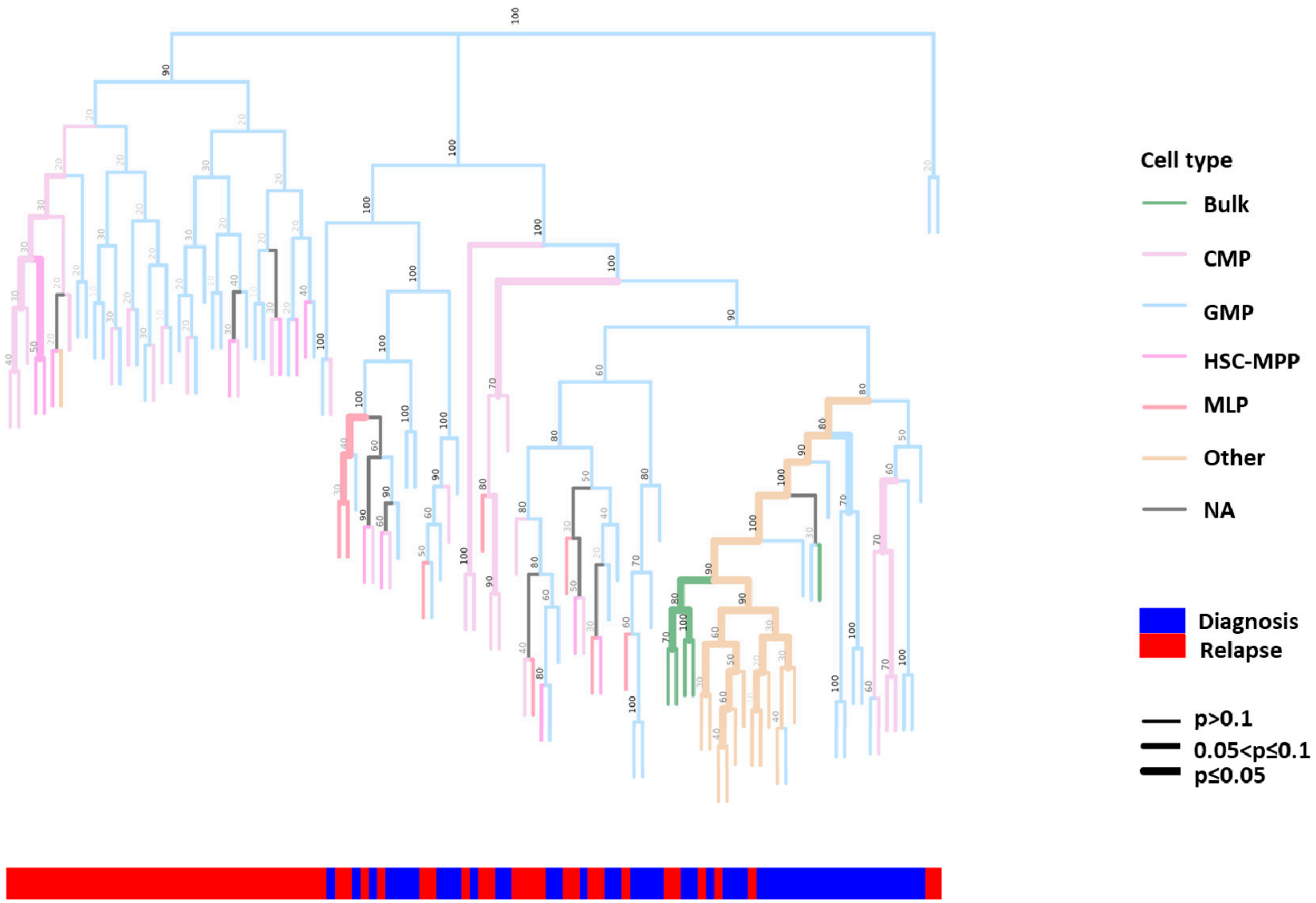

Cell type
Bulk
CMP
GMP
HSC-MPP
MLP
Other
NA
Diagnosis
Relapse
p>0.1
0.05<p≤0.1
p≤0.05

Figure S2. The phylogenetic tree of HSPC subpopulations derived from LCL230 patient at diagnosis and relapse.

Each tip represents a single cell or a single cell-derived subclone. The width of the colored branches represents the level of clustering significance (wider = lower *P* value). The root is calculated from an average signal of all HSC-MPP samples.

Boxplots show a comparison of cell division depth at different disease stages. Colors represent cell populations derived at: blue – diagnosis, red – relapse.

Colors represent: green – bulk; pink lace – CMP; light blue – GMP; light hot pink – HSC-MPP; orchid pink – MLP; light brown – other; grey – NA [no significant clustering ($P > 0.2$)]. The *P* value was calculated using the hypergeometric test.

HSC: hematopoietic stem cells; MPP: multipotent progenitor; CMP: common myeloid progenitor; MLP: multipotent lymphoid progenitor; GMP: granulocyte-monocyte progenitor

A

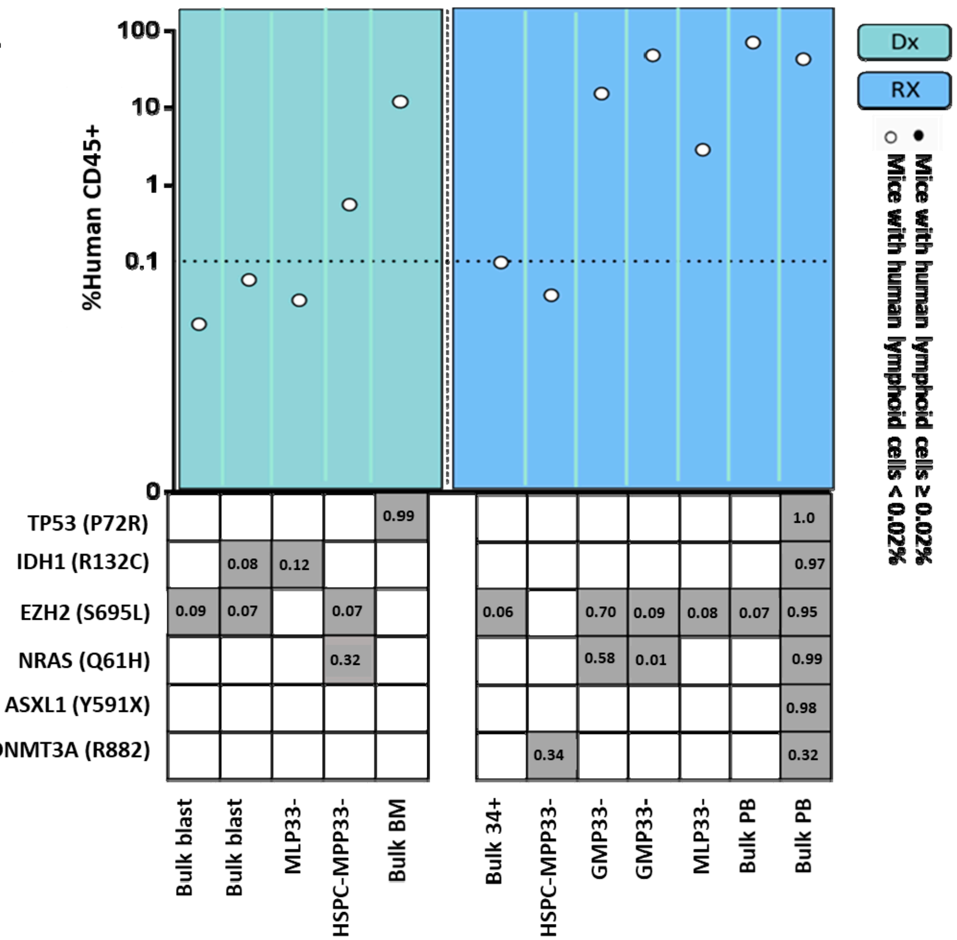

%Human CD45+
100
10
1
0.1
0
Dx
RX
Mice with human lymphoid cells ≥ 0.02%
Mice with human lymphoid cells < 0.02%
TP53 (P72R)
IDH1 (R132C)
EZH2 (S695L)
NRAS (Q61H)
ASXL1 (Y591X)
DNMT3A (R882)
0.99
1.0
0.08
0.12
0.97
0.09
0.07
0.07
0.06
0.70
0.09
0.08
0.07
0.95
0.32
0.58
0.01
0.99
0.98
0.34
0.32
Bulk blast
Bulk blast
MLP33-
HSPC-MPP33-
Bulk BM
Bulk 34+
HSPC-MPP33-
GMP33-
GMP33-
MLP33-
Bulk PB
Bulk PB

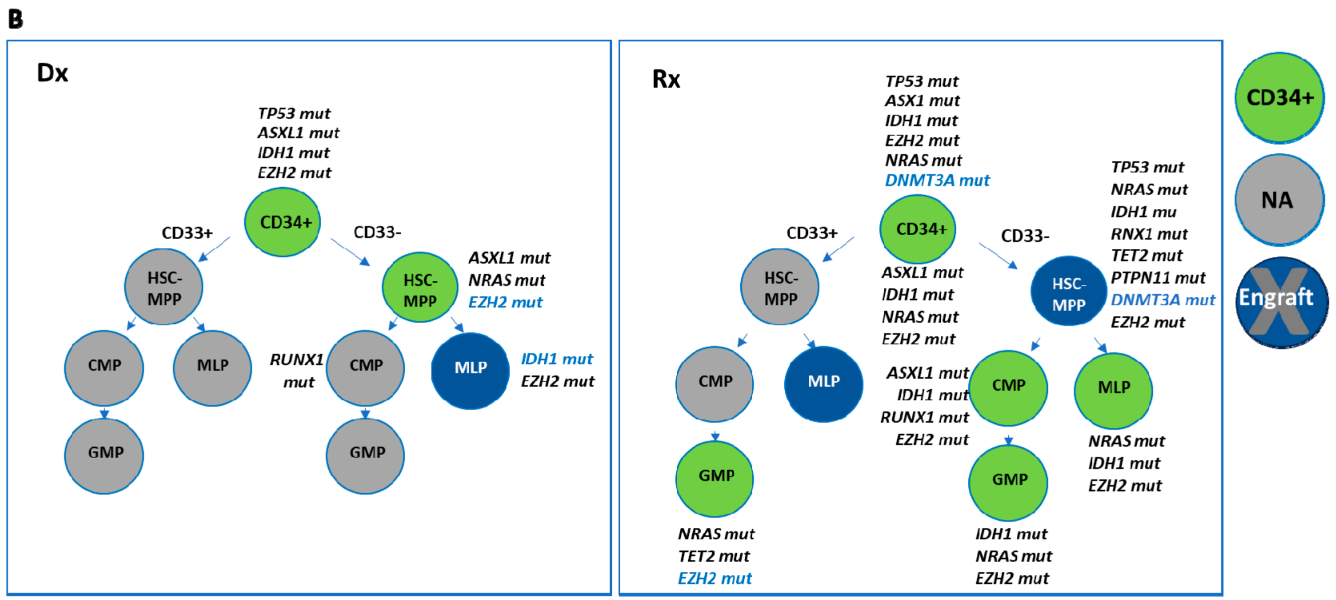

Figure S3. Evaluation of the HSPC leukemogenic potential for cells derived from patient LCL563.

(A) Bulk cells and HSPCs collected from sacrificed mice were analyzed for the presence of the hCD45$^+$ cell marker. The percentage of hCD45$^+$ cells out of the total number of mouse-derived cells is demonstrated. The dotted line indicates the generally accepted engraftment threshold set at 0.01. In this study focusing on single HSPCs and not on bulk cells, the threshold for engraftment detection was set at 0.02% of human CD45$^+$ (hCD45$^+$) cells. Cell engraftment of ≥5% is represented as black circles; cell engraftment of <5% is represented as white circles. The percentage of VAF of detected lesions in sorted hCD45$^+$ cells post-transplantation is indicated in the table depicting co-mutations.

(B) HSPC clonal composition at diagnosis and relapse. Green circles represent successful engraftment. Grey circles represent HSPCs unavailable for transplantation. Blue circles represent non-overt leukemic cell engraftment. AML-associated detected mutations are specified near each circle and colored according to their detection origin: black for mutations detected only in the bulk cells before injection; blue for mutations detected only after injection to mice.

Dx: diagnosis, Rx: relapse; HSC: hematopoietic stem cells; MPP: multipotent progenitor; CMP: common myeloid progenitor; MLP: multipotent lymphoid progenitor; GMP: granulocyte-monocyte progenitor; MEP: megakaryocyte-erythrocyte progenitor; BM: bone marrow; PB: peripheral blood

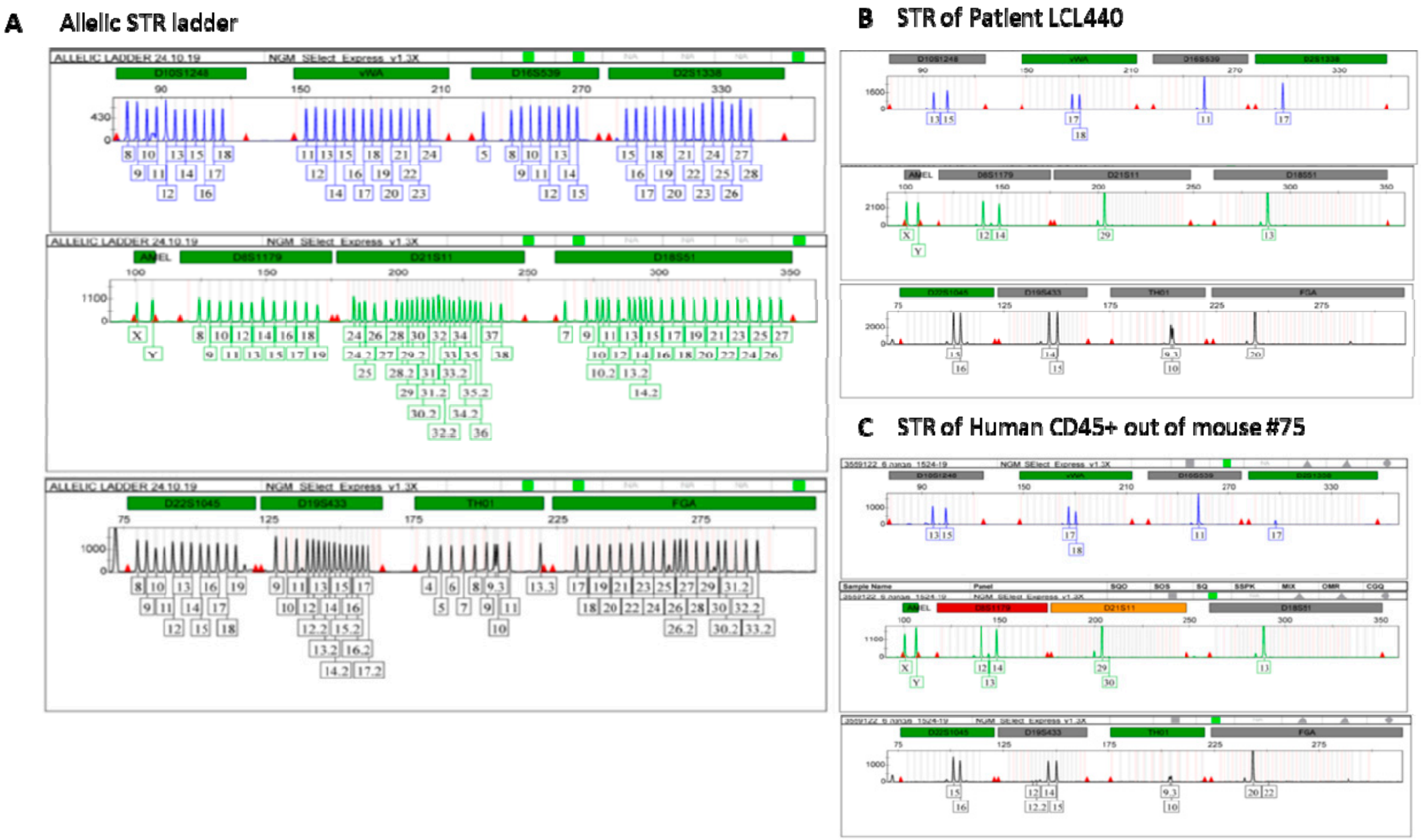

Figure S4. An example of the short tandem repeats analysis for cells derived from patient LCL440.

(**A**) Allelic short tandem repeats (STR) ladder

(**B**) Allelic STR analysis of a GMP33^{+} subpopulation

(**C**) Allelic STR analysis of an GMP33^{+} subpopulation engrafted to mouse #75 and then sorted for human CD45^{+} cells

Table S1. Summary of patient characteristics and clinical history

Characteristics at diagnosis														Treatment		Characteristics at relapse		
UPN	Gender	Age	WBC ($X10^3/\mu l$) at diagnosis	ELN risk stratification	% Blast cells in BM	Karyotype	FLT3-ITD (sizing)	FLT3-TKD	NPM1 mutation	DNMT3a mutation	IDH1 mutation	IDH2 mutation	Other mutations	Intensive chemotherapy (7+3)	Allogeneic BMT in first CR	Relapse	Time to relapse (days)	%Blast cells in BM
**AML patients**																		
LCL440	M	56	85.1	intermediate	75	NA	MUT AR=0.74	NA	MUT 432.5%	negative	NA	NA	NA	yes	no	yes	304	17
LCL465	M	21	45.6	high	48	t(9;22)	negative	NA	negative	negative	NA	NA	BCR/ABLWT1	yes	no	yes	286	35
LCL230	M	52	334.1	intermediate	80	NA	negative	NA	negative	NA	NA	NA	WT1/ABL	yes	no	yes	360	58
LCL563	F	66	2.13	adverse	40	del17p	negative	NA	negative	NA	yes R3132C	negative	NA	yes	yes	yes	746	84
LCL530	F	25	68.1	adverse	56	NA	MUT AR=1	negative	negative	NA	NA	NA	NA	yes	yes	yes	330	78
LCL582	F	21	3.05	favorable	40	t(8:21), del(9)	negative	NA	negative	NA	NA	NA	NA	yes	yes	yes	334	30
LCL248	M	34	76.8	favorable	59	inv-16	negative	NA	negative	NA	NA	NA	WT1/ABL	yes	yes	yes	60	12
LCL419	M	83	21.9	adverse	43	NA	MUT AR=0.67	NA	negative	negative	NA	NA	WT1/ABL	no	no	NA	NA	NA
LCL506	F	40	26.4	intermediate	61	Normal	negative	NA	negative	NA	negative	negative	NA	yes	yes	NA	NA	NA
LCL537	M	49	171.5	NA	80	NA	negative	negative	negative	NA	NA	NA	NA	yes	yes	no	NA	NA
LCL551	F	55	50.6	intermediate	74	Normal	MUT AR=0.67	negative	MUT 597%	positive R882H	NA	NA	NA	yes	yes	no	NA	NA
LCL554	F	27	1.03	NA	90	t(10:11), del(9)	negative	negative	negative	NA	NA	NA	NA	yes	yes	yes	291	46
LCL584	F	50	0.75	NA	64	NA	negative	negative	negative	NA	negative	positive c.515g>A 32%VAF	NA	yes	no	yes	NA	NA
LCL590	M	53	5.22	NA	76	NA	negative	negative	negative	NA	NA	NA	NA	yes	yes	NA	NA	NA
LCL591	F	30	16.6	adverse	73	-7	MUT AR=0.75	negative	negative	NA	NA	NA	NA	yes	yes	NA	NA	NA
**MDS patients**																		
LCL413	F	74	1.59	high	25	Normal	negative	negative	negative	negative	NA	NA	NA	no	no	no	NA	NA
LCL349	M	73	5.24	NA	8	Normal	negative	negative	negative	negative	NA	NA	NA	no	no	no	NA	NA
LCL341	F	80	3.71	NA	5	del5q	negative	negative	negative	negative	NA	NA	NA	no	no	no	NA	NA
LCL333	F	58	3.66	NA	5	inv9	negative	negative	negative	negative	NA	NA	NA	no	no	no	NA	NA
LCL321	F	68	3.1	low	12	del5q	negative	negative	negative	negative	NA	NA	NA	no	no	no	NA	NA
LCL101	F	71	5.0	NA	7	Normal	negative	negative	negative	negative	NA	NA	NA	no	no	no	NA	NA

UPN: unique patient number; WBC: white blood cells; FAB: French-American-British (FAB) classification; ELN: European LeukemiaNet; CR: complete remission; VAF: variant allelic frequency

Table S2. Immunophenotypes of healthy hematopoietic stem and progenitor cell subpopulations

Subpopulation	Immunophenotype
Hematopoietic stem cells (HSC)	$CD34^{+}$, $CD38^{-}$, $CD90^{+/-}$, $CD45RA^{-}$
Common myeloid progenitor (CMP)	$CD34^{+}$, $CD38^{+}$, $CD10^{-}$, $CD7^{-}$, $CD135(FLT)^{+}$, $CD45RA^{-}$
Granulocyte-monocyte progenitor (GMP)	$CD34^{+}$, $CD38^{+}$, $CD10^{-}$, $CD7^{-}$, $CD135(FLT)^{+}$, $CD45RA^{+}$
Megakaryocyte-erythrocyte progenitor (MEP)	$CD34^{+}$, $CD38^{+}$, $CD45RA^{-}$
Multipotent/common lymphoid progenitor (MLP/CLP)	$CD34^{+}$, $CD38^{-}$, $CD90^{+/-}$, $CD45RA^{+}$

Table S3. Mutation hotspot characterization

Gene	Mutation type	Region
ASXL1	Frameshift/nonsense/splice-site	exon 12
BCOR	Frameshift/nonsense/splice-site	whole gene
BCORL1	Frameshift/nonsense/splice-site	whole gene
BRAF	Missense	aa range p.590-615; G469
BRCC3	Frameshift/nonsense/splice-site	whole gene
CALR	Frameshift	exon 9
CBL	Missense	aa range p.345-434
CREBBP	Frameshift/nonsense/splice-site	whole gene
CSF1R	Missense	L301,Y969
DNMT3A	Frameshift/nonsense/splice-site	whole gene
DNMT3A	Missense	aa range p.292-350, p.482-614, p.634-912
EZH2	Frameshift/nonsense/splice-site	whole gene
EZH2	Missense	aa range p.617-732
GNAS	Missense	R201
GNB1	Missense	K57, I80
IDH1	Missense	R132
IDH2	Missense	R140, R172
JAK2	Missense/indel	V617F, aa range p.536-547
KDM6A	Frameshift/nonsense/splice-site	NM_021140
KRAS	Missense	G12, G13, Q61, A146
NPM1	Frameshift	exon 12
NRAS	Missense	G12, G13, Q61
PHF6	Frameshift/nonsense/splice-site	whole gene
PPM1D	Frameshift/nonsense	exon 5, 6
PRPF40B	Frameshift/nonsense/splice-site	whole gene
PTEN	Frameshift/nonsense/splice-site	whole gene
PTPN11	Missense	aa range p.58-76, p.491-510
RAD21	Frameshift/nonsense/splice-site	whole gene
SF1	Frameshift/nonsense/splice-site	whole gene
SF3A1	Frameshift/nonsense/splice-site	whole gene
SF3B1	Missense	aa range p.529-1201
SMC1A	Missense	R96, R586

SMC3	Frameshift/nonsense/splice-site	whole gene
SRSF2	Missense/deletion	P95
STAG2	Frameshift/nonsense/splice-site	whole gene
STAT3	Missense	aa range p.580-670
TET2	Frameshift/nonsense/splice-site	whole gene
TET2	Missense	p.1104-1481, p.1843-2002
TP53	Frameshift/nonsense/splice-site	whole gene
TP53	Missense	aa range p.95-288, P72, R337
U2AF1	Missense	S34, R156, Q157
U2AF2	Missense	aa range p.149-231, p.259-337, p.381-462
ZRSR2	Frameshift/nonsense/splice-site	whole gene
CEBPA	Frameshift/nonsense/splice-site	whole gene
RUNX1	Frameshift/nonsense/splice-site	whole gene
RUNX1	Missense	aa range p.100-440
SETBP1	Missense	868, 870
FLT3	In-frame	580-820
FLT3	Missense	835, 839, 841
KIT	Frameshift	416-418
KIT	Missense	816,419
KMT2D	Frameshift/nonsense/splice-site	whole gene
NF1	Frameshift/nonsense/splice-site	whole gene
WT1	Frameshift/nonsense/splice-site	whole gene
